# Multinucleated cell angiohistiocytoma: independent entity or variant of dermatofibroma? Rosettes and shiny white lines as new dermoscopic findings^؆^

**DOI:** 10.1016/j.abd.2025.501183

**Published:** 2025-08-15

**Authors:** Sara Becerril-Andrés, Eduardo de la Rosa-Fernández, Jesús del Pozo-Losada

**Affiliations:** a Dermatology Department, Hospital Universitario de La Plana, Villarreal, Castellón, Spain; b Dermatology Department, Hospital Universitario Nuestra Señora de Candelaria, Santa Cruz de Tenerife, Spain; c Dermatology Department, Complejo Hospitalario Universitario A Coruña, A Coruña, Spain

*Dear Editor,*

Multinucleated Cell Angiohistiocytoma (MCA) is an unusual benign vascular and fibro-histiocytic proliferation, predominantly affecting women aged 40 to 70 years.^1^ Clinically, it presents as asymptomatic violaceous or brownish papules, either solitary or clustered, primarily located on the dorsal hands and extremities, although generalized forms have also been described.^1^, ^2^ These lesions may resemble annular granuloma, sarcoidosis, Kaposi sarcoma, lichen planus, or dermatofibroma.^1^

Traditionally, MCA has been described as a vascular cutaneous tumor. Although its pathogenesis remains unclear, recent observations suggest it may instead represent a fibrohistiocytic process or a chronic inflammatory condition with fibrohistiocytic and vascular hyperplasia.^2^, ^3^ Controversial hypotheses propose MCA as a particular variant of dermatofibroma, while others argue that it is a distinct entity with unique clinical, histological, and immunohistochemical characteristics.^2^, ^4^ The influence of female hormones on its pathogenesis has also been suggested, supported by the identification of estrogen receptor alpha in interstitial and multinucleated cells, which may explain its higher prevalence in women.^5^

Histologically, MCA is characterized by a proliferation of capillaries and venules in the superficial and mid-dermis, accompanied by prominent endothelial cells. Thickened collagen fibers parallel to the epidermis and frequent acanthosis of the epidermis are also observed. The most striking finding, although not pathognomonic, is the presence of multinucleated giant cells, typically positive for vimentin and factor XIIIa, with variable positivity for CD68. These cells are also found in other fibrohistiocytic proliferations, including dermatofibromas, angiofibromas, and nasal fibrous papules, with secondary vascular proliferation. This supports the hypothesis of MCA being a fibro-histiocytic process.^1^

Few studies have addressed the dermoscopy of MCA. Valerón-Almazán et al. were the first to describe its dermoscopic features, identifying three main structural patterns: diffuse reddish areas (corresponding to dilated vessels), whitish spots (associated with collagen thickening), and fine reticulated peripheral areas (likely representing melanin in epidermal ridges).^6^ Despite the increasing number of reported cases, we found no additional dermoscopic descriptions of MCA in the literature.

We present four histologically confirmed cases of MCA with dermoscopic descriptions, summarized in Table 1. Three patients were female, and the age range was 60 to 65 years. Histological examination revealed characteristic features of MCA, including multinucleated giant cells, thickened collagen fibers parallel to the epidermis, and proliferation of thickened capillaries and small venules in the superficial and mid-dermis (Fig. 1). Immunohistochemistry was not performed in our cases, as the histological images and the clinicopathological correlation were consistent with the diagnosis of multinucleated cell angiohistiocytoma. All cases presented clinically similar lesions: small flat violaceous or brownish papules located on the dorsal hands (Fig. 2). The dermoscopic structures observed included rosettes, shiny white lines, pink, whitish, and homogeneous brown areas, homogeneous brown periphery, vascular dots, and whitish reticulated patterns (Fig. 3). All these structures, except for rosettes and shiny white lines, were previously described by Zeballos et al. for dermatofibromas.^7^ Under polarized light dermoscopy, three cases showed rosettes and three cases exhibited shiny white lines (two cases had both structures), findings not previously described in MCA.

**Table 1:** Summary of clinical and dermoscopic characteristics.

		Case A (Figs. 2A, 3A)	Case B (Figs. 2B, 3B)	Case C (Figs. 2C, 3C)	Case D (Figs. 2D, 3D)
**Sex**		Female	Female	Female	Male
**Age (years)**		65	65	60	61
**Location**		Dorsal left hand	Dorsal left hand	Dorsum of both hands	Dorsal right hand
**Evolution (years)**		3	3	5	7
**Dermoscopic structures (Fig.**3**)**	Rosettes (→)	+	+	+	–
	Shiny white lines (=)	+	+	–	+
	Whitish reticulated pattern (×)	+	+	–	–
	Vascular dots (>)	+	+	–	+
	Pink diffuse areas	+	+	+	+
	Whitish diffuse areas	+	+	+	–
	Brown diffuse areas	+	+	+	–
	Homogeneous brown periphery (*)	+	+	+	–

**Fig. 1:**
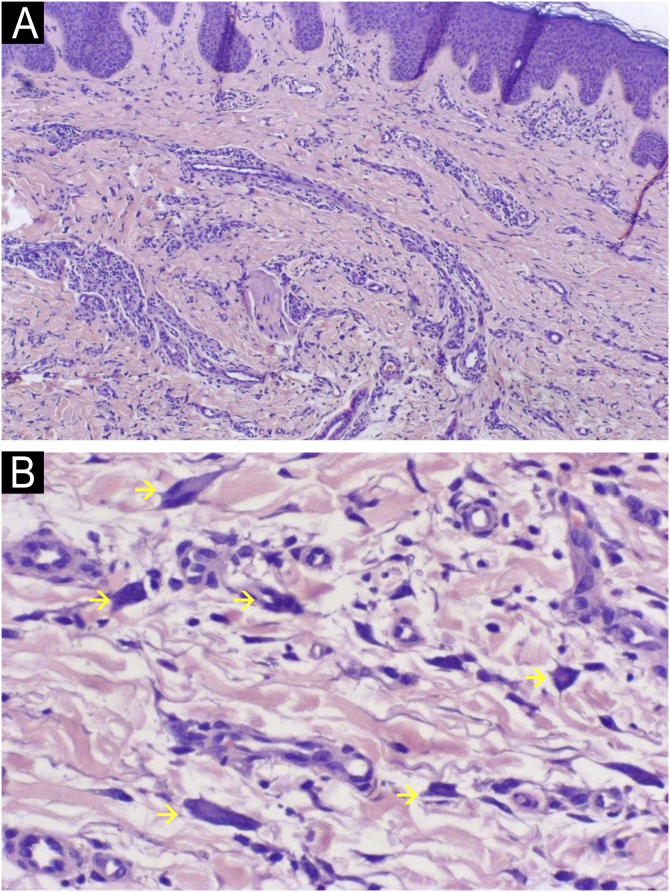
Histological images. (A) Low magnification view showing proliferation of thickened capillaries and small venules within a collagenous fibrotic stroma in the superficial and mid dermis (Hematoxylin & eosin, ×100). (B) Higher magnification picture showing the multinucleate cells (yellow arrows) (Hematoxylin & eosin, ×400).

**Fig. 2:**
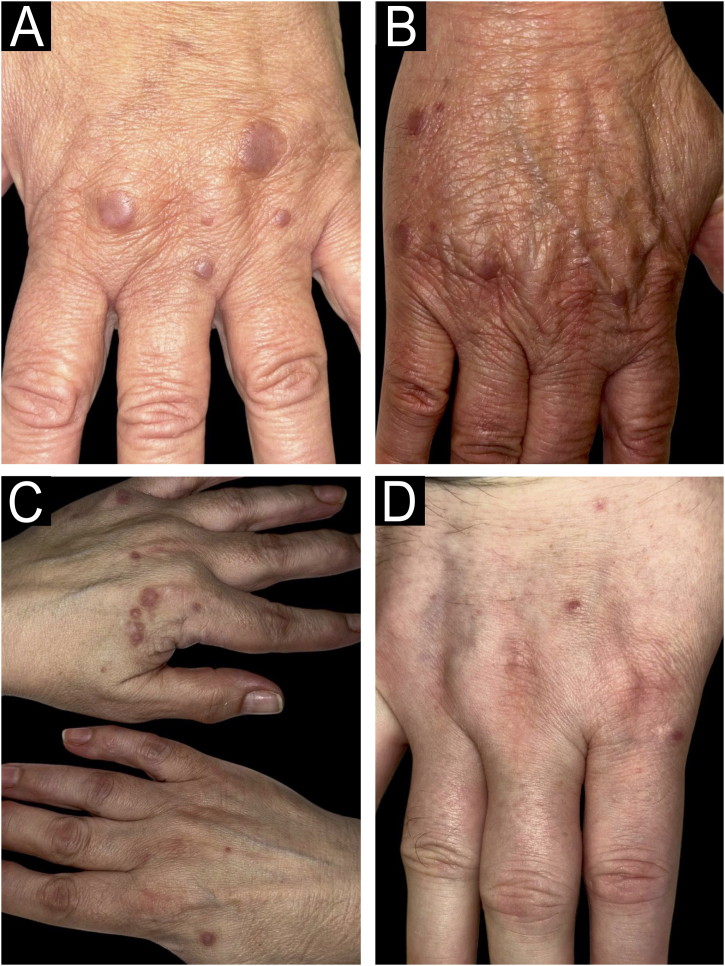
Clinical images. Small flat violaceous or brownish papules clustered on the dorsal aspect of the hands.

**Fig. 3:**
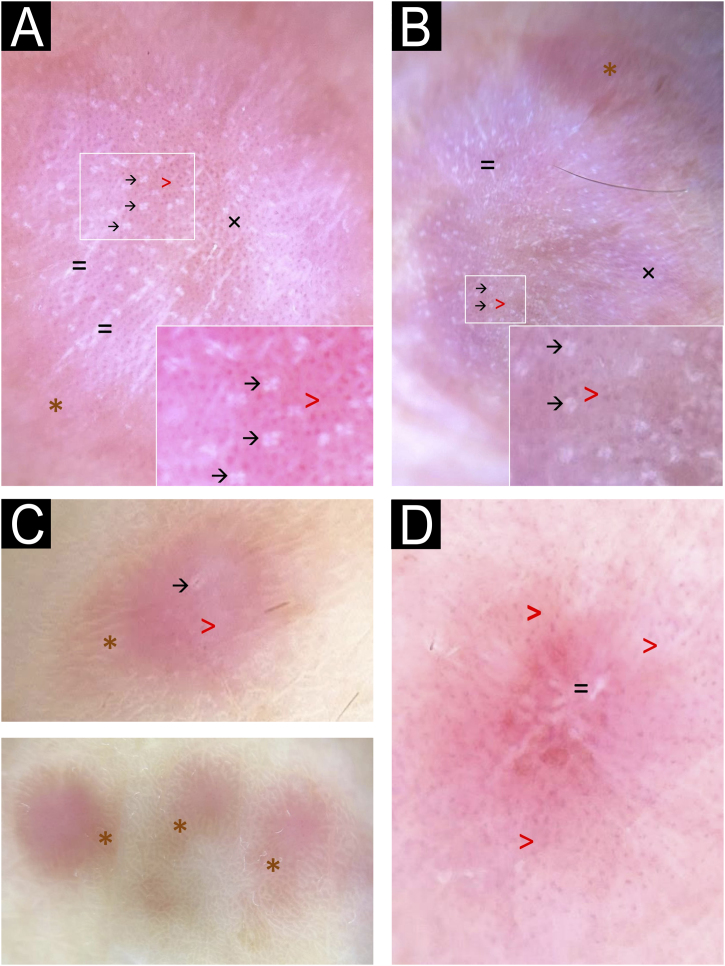
Dermoscopic images. Observed structures include homogeneous brown periphery (*), vascular dots (>), rosettes (→), shiny white lines (=), and whitish reticulated pattern (×). Pink, brown, and/or whitish diffuse areas were observed in all cases, with some lesions showing a homogeneous brown periphery (*). Regular vascular dots (>) were seen in all cases. Panels A and B highlighted multiple rosettes with a consistent distribution and orientation, whereas panel C revealed a single rosette at the lesion's center (→). Panels A and B also showed multiple shiny white lines diffusely distributed, with some localized at the lesion's center in panel B (=). Whitish reticulated areas (×) were also observed in panels A and B.

Rosettes are a type of shiny white structure visible only under polarized light dermoscopy, characterized by four bright white dots arranged like a four-leaf clover.^8^, ^9^ Unlike shiny lines and areas, likely caused by fibrotic dermal changes, the exact histopathological correlation of rosettes remains unknown. Smaller rosettes (0.1–0.2 mm) are believed to result primarily from polarizing keratin material at the infundibular level in appendageal openings, while larger rosettes (0.3–0.5 mm) may be due to concentric perifollicular fibrosis. Historically, rosettes were considered common in actinic keratoses and squamous cell carcinoma. However, they have been observed in multiple non-keratinocytes and inflammatory lesions, including dermatofibromas, though very infrequently.^8^, ^9^

Shiny white lines can appear orthogonal, parallel, centrifugal, or more disorganized and are primarily caused by the polarization of thickened hyaline fibrous bundles. They have been frequently described in dermatofibromas and scars, as well as in other lesions such as melanoma, Spitz nevus, basal cell carcinoma, and porokeratosis.^10^

We present new dermoscopic findings for MCA, highlighting the importance of this tool in characterizing the entity. Although rosettes and other dermoscopic structures are not specific to any condition, their presence can assist in the differential diagnosis of other conditions that clinically mimic MCA, such as Kaposi sarcoma, lichen planus, sarcoidosis, annular granuloma, or viral warts.^6^ We observed significant dermoscopic similarities between MCA and dermatofibroma, supporting the hypothesis that MCA may indeed represent a histiocytic-origin entity.

## Research data availability

Does not apply.

## Scientific Associate Editor

Hiram Larangeira de Almeida Jr.

## Authors' contributions

Sara Becerril Andrés: Conceptualized the study; conducted the literature review, collected and analyzed the clinical data, and wrote the original draft of the manuscript.

Eduardo de la Rosa Fernández: Assisted in the interpretation of findings, and reviewed the final version of the manuscript for approval.

Jesús del Pozo Losada: Contributed to the clinical management of the patient, provided expert input on the discussion section, and reviewed the final version of the manuscript for approval.

## Financial support

None declared.

## Conflicts of interest

None declared.

## References

[1] Grgurich E., Quinn K., Oram C., McClain R., Lountzis N. (2019). Multinucleate cell angiohistiocytoma: case report and literature review. J Cutan Pathol.

[2] Jia Q.N., Qiao J., Qu T. (2021). Generalized multinucleate cell angiohistiocytoma with possible origin from fibroblasts: a clinicopathological study of 15 cases. J Dermatol.

[3] Frew J.W. (2015). Multinucleate cell angiohistiocytoma: clinicopathological correlation of 142 cases with insights into etiology and pathogenesis. Am J Dermatopathol.

[4] Roy S.F., Dong D., Myung P., McNiff J.M. (2019). Multinucleate cell angiohistiocytoma: a clinicopathologic study of 62 cases and proposed diagnostic criteria. J Cutan Pathol.

[5] Cesinaro A.M., Roncati L., Maiorana A. (2010). Estrogen receptor alpha overexpression in multinucleate cell angiohistiocytoma: new insights into the pathogenesis of a reactive process. Am J Dermatopathol.

[6] Valerón-Almazán P., Dehesa L., Santana N., Vilar J., Carretero G. (2011). Hallazgos dermatoscópicos del angiohistiocitoma de células multinucleadas: ¿una variante de dermatofibroma? [Dermoscopic features of multinucleate cell angiohistiocytoma: a variant of dermatofibroma?]. Actas Dermosifiliogr.

[7] Zaballos P., Puig S., Llambrich A., Malvehy J. (2008). Dermoscopy of dermatofibromas: a prospective morphological study of 412 cases. Arch Dermatol.

[8] Alorainy M., Buchanan K., Nussinow T., Rabinowitz J.B., Cyr P., Seiverling E.V. (2024). A systematic review of diagnoses with rosettes under dermoscopy. Dermatol Pract Concept..

[9] Haspeslagh M., Noë M., De Wispelaere I., Degryse N., Vossaert K., Lanssens S. (2016). Rosettes and other white shiny structures in polarized dermoscopy: histological correlate and optical explanation. J Eur Acad Dermatol Venereol.

[10] Balagula Y., Braun R.P., Rabinovitz H.S., Dusza S.W., Scope A., Liebman T.N. (2012). The significance of crystalline/chrysalis structures in the diagnosis of melanocytic and nonmelanocytic lesions. J Am Acad Dermatol.

